# Heat-Labile Enterotoxin: Beyond G_M1_ Binding

**DOI:** 10.3390/toxins2061445

**Published:** 2010-06-14

**Authors:** Benjamin Mudrak, Meta J. Kuehn

**Affiliations:** 1 Department of Molecular Genetics and Microbiology, Duke University Medical Center, Durham, NC 27710, USA; Email: bm36@duke.edu; 2 Department of Biochemistry, Duke University Medical Center, Durham, NC 27710, USA

**Keywords:** heat-labile enterotoxin, ETEC, G_M1_, lipopolysaccharide, blood antigen

## Abstract

Enterotoxigenic *Escherichia coli* (ETEC) is a significant source of morbidity and mortality worldwide. One major virulence factor released by ETEC is the heat-labile enterotoxin LT, which is structurally and functionally similar to cholera toxin. LT consists of five B subunits carrying a single catalytically active A subunit. LTB binds the monosialoganglioside G_M1_, the toxin’s host receptor, but interactions with A-type blood sugars and *E. coli* lipopolysaccharide have also been identified within the past decade. Here, we review the regulation, assembly, and binding properties of the LT B-subunit pentamer and discuss the possible roles of its numerous molecular interactions.

## 1. Introduction

### 1.1. Enterotoxigenic *Escherichia coli*

Enterotoxigenic *Escherichia coli* (ETEC) causes a form of intestinal disease termed traveler’s diarrhea, which affects nearly every population worldwide. In general, this malady is self-limiting in a healthy adult, although antibiotics are often prescribed [1]. In addition to traveler’s diarrhea, ETEC can cause disease symptoms clinically indistinguishable from cholera caused by *Vibrio cholerae* [2,3]. ETEC is endemic in many developing countries, including Mexico and Bangladesh, and is frequently encountered by tourists, members of the military, or other visitors [1]. Because of poor surveillance, mortality due to ETEC is difficult to estimate, but there are believed to be at least 400,000 ETEC-related deaths in children under the age of 5 each year, with countless others likely classified simply as death due to diarrhea [4]. Human ETEC strains are closely related to numerous isolates taken from pigs suffering from diarrhea, with both types of ETEC sharing a number of pathogenic features and virulence factors, including heat-labile enterotoxin (LT). However, human and porcine ETECs show strong host preferences that are understood to be due to the expression of fimbriae with distinct tropisms [5].

A large of number of disease-causing ETEC strains have been isolated from patients, with over 70 identified O-antigen serogroups, along with over 25 recognized variants of adhesive fimbriae and a pair of enterotoxin families carried by ETEC at varying frequencies (see below) [6]. Extrachromosomal plasmids carrying virulence determinants are present in the vast majority of ETEC strains, providing the bacteria with the genes to produce toxins and fimbriae as well as the potential to mobilize these genes, creating new enterotoxigenic strains [7]. Attempts to generate an effective vaccine against ETEC, particularly for young children, have mostly met with failure due to the highly variable nature of the antigens present amongst strains [8].

### 1.2. Enterotoxins produced by ETEC

By convention, ETEC strains are classified based on their expression of LT (described in detail in this review), a heat-stable enterotoxin (ST), or both [9]. ST molecules are small peptides that mimic the native intestinal hormone guanylin and activate guanylyl cyclase [10], and LT was originally named to describe a heat-sensitive enterotoxigenic factor distinguishable from the heat stability of ST. A second class of LT molecules, termed LT-II, also exists (the prototypical class of LT is sometimes called LT-I). While structurally similar, the B subunit of LT-II shares little sequence similarity to LT-I, and strains expressing LT-II are rarely isolated from human patients [11]. Unless otherwise stated, ‘LT’ will refer solely to human LT-I in this review.

Incubation of LT at 70 °C for 10 minutes is sufficient to destroy its activity [12], whereas boiling does not inactivate ST. ST and LT both serve to disrupt the balance of electrolytes in the intestine, causing the diarrhea associated with ETEC infection. Out of 798 LT isolates surveyed in 1997, 196 (25%) expressed LT, 376 (46%) expressed ST, and 231 (29%) carried both toxins [9]. Thus, over half of all ETEC isolates express LT. The activity of LT promotes the adherence of ETEC cells to enterocytes *in vitro* [13], and expression of LT is required for ETEC to colonize the mouse intestine and to cause disease symptoms in gnotobiotic piglets [14,15]. Thus, while all ETEC isolates have the potential to cause diarrhea, those expressing LT may have an advantage in terms of colonization.

**Figure 1:**
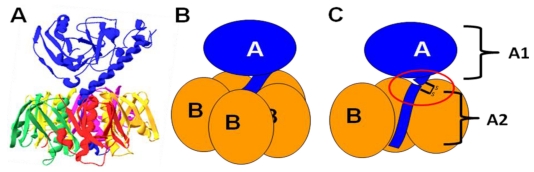
The structure and subunit organization of LT. (**A**) The crystal structure of LT holotoxin, generated using Swiss-PDB viewer (version 4.0.1) using PDB number 1LTA. The globular A1 fragment and the helical A2 peptide can be seen in blue, along with a ring of five B subunits. (**B**) A schematic of the subunit organization of LT. (**C**) A schematic of LT showing a cutaway view of the toxin’s central core. The location of the site of proteolytic processing (“nicking”), which is subtended by a disulfide bond, is circled. Nicking occurs after secretion of the toxin, and the disulfide is reduced inside the host cell, releasing the catalytically active A1 fragment.

In terms of both structure and function, LT is closely related to cholera toxin (CT) from *V. cholerae*. Like CT, LT is a multimeric AB_5_ toxin, composed of a single A subunit (LTA) associated with a ring of five B subunits (LTB) [16]. Figure 1 shows the structure and subunit organization of LT. Heat treatment of the toxin breaks down the pentameric LTB ring into monomers, releasing LTA. While catalytic activity is present in free LTA, the LTB pentamer is required for entry into cells of the intestinal epithelium, and disruption of the holotoxin thereby prevents intoxication of host cells [17]. 

### 1.3. Catalytic activity of LT

Early research efforts determined that the catalytic activity of LT is identical to that of CT, which had already been characterized. Specifically, LTA ADP-ribosylates an arginine residue in the α subunit of the host G_s_ protein [18]. This modification leads to inhibition of the GTPase activity of G_sα_, rendering adenylate cyclase constitutively active. Increased levels of cyclic AMP (cAMP) in the host cell open several channels, including the cystic fibrosis transmembrane receptor, resulting in the loss of fluid and electrolytes into the intestinal lumen [19]. cAMP-dependent cellular events also lead to the downregulation of antimicrobial peptides in the intestine [20]. LTA displays limited auto-ADP-ribosylation activity, especially when overexpressed, but this reaction does not seem to influence its function [21].

During activation, LTA is proteolytically cleaved, releasing a helical *C*-terminal fragment termed A2; the remaining A1 polypeptide is responsible for the observed toxic effects. This cleavage event, termed “nicking,” is not required for the toxic effects of LT, but mutants that are unable to be nicked demonstrate a markedly delayed effect in cell culture, and nicking doubles the toxin’s ADP-ribosyltransferase activity *in vitro* [21]. Trypsin is able to cleave LTA into A1 and A2 *in vitro*, but the identity of the protease(s) involved in this activation step *in vivo* is not known [22]. In addition to this cleavage event, a disulfide bond linking A1 and A2 is also reduced after entry into the host cell in order to completely separate the two fragments [23]. This disulfide bond is not essential to holotoxin formation, but mutation of the cysteine residues involved makes LTA more sensitive to degradation by proteases and generates a significant lag in cAMP production in cultured intestinal cells compared to wild-type toxin [24].

### 1.4. Homology to CT

LT is encoded by a two-gene operon, with the gene for LTA (*eltA*) overlapping with the start of the gene for LTB (*eltB*) by four nucleotides [25]. These genes have been referred to as *etxAB* or *toxAB* in some older studies [26] but will be called *eltAB* here, following recent convention. Given the identical subunit structure and catalytic activity, it is perhaps unsurprising that the nucleotide sequences of the genes for CT and LT are highly similar. The *eltAB* operon demonstrates approximately 78% overall nucleotide sequence identity to the *ctxAB* operon coding for CT [27]. While the DNA sequences display a fairly uniform similarity throughout, the amino acid sequences of the subunits of CT and LT contain “hotspots” of greater divergence. The most divergent regions include the signal sequences targeting the subunits to the periplasm (see below) and the A2 peptide [27]. Most relevant to the ligand binding properties described in this review, the two mature B subunits share 83% amino acid identity [28]. 

Sequence analysis indicates that the genes encoding LT were acquired by horizontal transfer from *V. cholerae* around 130 million years ago, long after *V. cholerae* and *E. coli* diverged as species [29]. In ETEC, *eltAB* is found on an extrachromosomal virulence plasmid called pEnt [30]. When surveying ETEC strains, these genes were found to be flanked by approximately 250 base pairs of conserved sequence, often followed by partial or intact insertion sequence (IS) elements [30]. IS elements similar to those flanking *eltAB* are frequently observed next to genes encoding host-binding fimbriae, suggesting a general mechanism for the transfer of virulence-related genes. In addition, entire pEnt plasmids can be transferred to non-pathogenic strains of *E. coli*, rendering them toxigenic [31].

## 2. Regulation of LT Production

### 2.1. Growth conditions inducing the release of LT

Unlike the extensively studied regulatory network controlling CT production [32], less is known about the transcriptional regulation of *eltAB*. Alteration of ETEC’s growth conditions revealed that a pH of 7.5–8.0 maximizes the amount of LT released; moreover, LT is not produced in detectable quantities at temperatures lower than 26 °C, with toxin production increasing along with temperature and reaching a peak at 37 °C [33,34]. It has also been observed that the addition of glucose to the growth media induces the release of LT [33,35]. In addition, oxygen levels and osmolarity were found to influence toxin expression. Specifically, microaerophilic conditions and increased salt concentrations (>171 mM) both promote the production of LT [36]. In general, conditions that mimic those found in the human small intestine, where LT is expected to play a role in ETEC-associated pathogenesis, are optimal for production of the toxin. 

The presence of short-chain fatty acids (particularly those with carbon chain lengths between three and eight) in the growth media impairs the production of LT [37]. Short-chain fatty acids are produced in relatively large quantities in the colon, where they stimulate fluid absorption in a cAMP-independent manner [38]. Thus, they may serve to signal ETEC that it has reached the large intestine and no longer needs to produce high quantities of LT, having passed its target, the ileum. Additionally, the presence of short-chain fatty acids may indicate to ETEC that conditions in the gut favor the absorption of liquid by the colonic epithelium and that the production of LT would not be effective. 

While many of these conditions (e.g., higher temperature) consistently induce LT production, it should be noted that not all ETEC isolates are identical in the levels of LT that they produce. A study of 26 LT-positive Brazilian ETEC isolates found that the secreted levels of the toxin varied almost 50-fold [39]. While the amount of LT produced correlated well with fluid accumulation in a rabbit ileal loop model, LT-hyperproducing strains were isolated from diarrheic patients and asymptomatic patients with equal frequencies in that study. Although there is no way to tell whether the production of LT measured *in vitro* recapitulated the strains’ phenotypes *in vivo*, a careful study of *eltB* expression in diarrheagenic ETEC isolates both immediately after collection and later, after freezedown and storage, indicated little change due to *in vitro* growth [40]. Therefore, while LT certainly contributes to disease symptoms, the regulation of LT production may occur differently in individual ETEC isolates.

### 2.2. Regulation by H-NS

Apart from characterization of the growth conditions favoring LT production, little was known about the transcriptional regulation of *eltAB* for decades. The conserved region upstream of *eltA* contains a strong consensus promoter, and the region downstream of *eltB* contains a probable transcriptional terminator, indicating that these genes are transcribed as a single message [30,41]. In 1998, Trachman and Maas noted that deletion of the *eltA* gene elevates the levels of mRNA transcribed from the operon’s promoter, which they determined to be due to lack of a downstream regulatory element bound by the nucleoid-associated protein H-NS [42]. In a strain lacking H-NS, *eltAB* is not repressed at low temperatures [42]. Later work by another group uncovered two distinct but cooperative binding sites for H-NS positioned in the region downstream of the promoter [43]. These sites are found at regions of DNA predicted to have significant curvature, a feature of H-NS binding elements [43]. While there is interplay between the effects of temperature and osmolarity, the regulation of *eltAB* in response to salt concentration is independent of H-NS and its regulatory element [36]. Therefore, H-NS is responsible for temperature regulation of LT, but it is unclear which bacterial factors are able to sense osmotic conditions and short-chain fatty acids.

### 2.3. Feedback from cAMP

Another important regulator of the transcription of *eltAB* is cAMP, an end product of LT’s catalytic activity. As described above, the catalytic activity of LTA causes an increase in cAMP levels in host cells. Some of this cAMP is shed into the intestinal lumen after treatment with LT [44], and there is evidence that exogenous cAMP can be sensed by ETEC. Namely, conditioned supernatant from ETEC-treated intestinal epithelial cells (or pure cAMP, the active component) stimulates the LT-dependent adherence of ETEC to enterocytes *in vitro* [13]. It was later determined that bacterial cAMP receptor protein (CRP) suppresses the transcription of *eltAB* by binding to an operator overlapping the σ^70^ consensus binding site [45]. While seemingly contradictory, these results may define a feedback loop, whereby the production of LT can be downregulated once a certain amount of cAMP has been released by the cells of the intestinal epithelium. 

**Figure 2:**
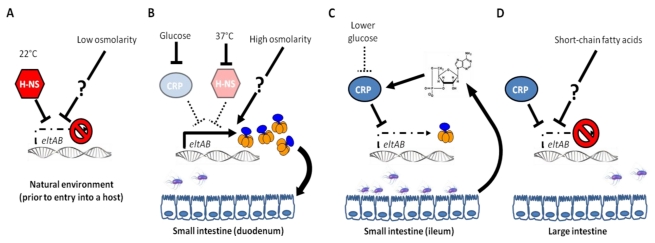
A model of the regulation of *eltAB* during infection. (**A**) In an environmental reservoir at ambient temperature, low osmolarity and H-NS act to repress the *eltAB* promoter. No LT is produced. (**B**) Upon entry into the host intestine, free glucose and higher temperature de-repress the *eltAB* operon by inhibiting cAMP receptor protein (CRP) and H-NS, respectively. High levels of LT are produced. High osmolarity may also contribute to the production of LT by an unknown mechanism. (**C**) ETEC reaches the ileum, where it begins to multiply, adhering to intestinal cells due to the activity of LT. In the ileum, glucose concentrations are lower due to intestinal absorption, and significant amounts of cAMP have been released by host cells in response to LT. These signals combine to repress *eltAB* via CRP. Little new LT is produced. (**D**) In the large intestine, CRP and short-chain fatty acids produced by commensal bacteria fully repress *eltAB*. The mechanism of repression by short-chain fatty acids is unknown. ETEC shuts off the production of LT in preparation to exit the host.

The identification of CRP as a negative regulator of LT synthesis also served to explain earlier observations regarding glucose. Glucose inhibits bacterial cAMP production, lowering levels of active CRP and thereby upregulating LT production [45]. As glucose is absorbed by the small intestine, ETEC is likely to encounter higher concentrations of the sugar in early sections of the gut [46]. Thus, early exposure to glucose may induce the production of LT to aid in adherence to the ileum, where most ETEC cells are found in a mouse model of colonization [14]. Interestingly, ST production is stimulated by CRP [45], suggesting that ETEC may predominantly produce one toxin at a time during infection. Together, these results leave us with a model whereby ETEC senses conditions that identify the small intestine and, in turn, upregulates the production of LT (Figure 2). Once ETEC has reached the large intestine, LT synthesis shuts off as the bacteria prepare to exit the host.

## 3. Holotoxin Assembly

### 3.1. Periplasmic localization

Both LTA and LTB feature signal sequences directing them to the periplasm of *E. coli* through the Sec translocation machinery. For LTA, this signal is 18 amino acids in length, while LTB contains a 21-amino acid signal peptide [47,48]. Each peptide sequence contains positive charges at the beginning, a core of hydrophobic residues, and a tyrosine or histidine residue near the cleavage site, in line with typical *sec*-dependent signals. For many years, the periplasm was thought to be the terminal destination of LT [49], although it is now known that it remains periplasmic in laboratory strains of *E. coli* because they do not express a functional type II secretion system (see below).

Once LTA and LTB subunits reach the periplasm, they spontaneously assemble into holotoxins containing one A subunit and five B subunits. Pentamers form quite rapidly; within one minute, 80% of newly transported LTB has formed into pentamers, about half of which carry LTA [50]. LTA is actually produced in slight excess, such that around 1.5 LTA subunits are found for every 5 LTB subunits in the periplasm. Pulse-chase studies of LTA determined that 90% of periplasmic LTA is associated with B-subunit pentamers at any one time, but half of newly transported LTA does not form holotoxins [50]. Thus, it is likely that LTA is made in excess because it can be easily degraded in the periplasm before it can assemble into holotoxins. Fully formed holotoxin is remarkably stable, remaining assembled at any pH between 2.0 and 11.0 [51]. Moreover, while exposure to strong acidic conditions dissociates the B pentamer, free monomers re-oligomerize upon neutralization with no additional factors present [16].

### 3.2. Factors influencing oligomerization

Much of what is required to form LT holotoxins exists within the amino acid sequences of the subunits themselves. To date, the only prokaryotic factor known to be required for the formation of LT (and CT) is DsbA, a periplasmic disulfide oxidoreductase. LTB contains a single disulfide bridge linking Cys-9 and Cys-86. Disruption of this bond with a reducing agent such as dithiothreitol prevents reassembly of LT after dissociation in acid, and a transposon insertion in the *V. cholerae* homolog of *dsbA* was reported to abolish the heterologous production of LT [16,52]. LTA also features an important disulfide bond linking the A1 and A2 fragments at Cys-187 and Cys-199, the formation of which is presumably catalyzed by DsbA. Other studies of the mechanism behind the acid dissociation of LT and its subsequent reassembly under neutral conditions showed that isomerization of Pro-93 in the B subunit blocks oligomerization [53]. The results of that study suggest that proper formation of LT requires the activity of a peptidyl proline isomerase *in vivo*, but this hypothesis has yet to be tested.

While free B subunits are intrinsically capable of assembling pentamers, the presence of LTA enhances pentamer formation. Consequently, a larger amount of oligomerized LTB can be found when LTA is co-expressed [16]. This effect is due to the A2 peptide, as deletion of the last 14 amino acids from LTA significantly slows the formation of B-subunit pentamers [54]. Interestingly, the last four amino acids of LTA (Arg-Asp-Glu-Leu) may specifically be critical for anchoring LTA in the newly formed pentamer. LTA mutants lacking these residues are able to readily induce LTB oligomerization, but are not found with the assembled pentamers [54]. Thus, the “cargo” subunit of LT (LTA) also lends a hand in the assembly of native toxin.

B-subunit mutants that are defective in pentamer assembly have also been isolated. LTB in which the final amino acid residues, Ser-Met-Glu-Asn, have been replaced with Gly-Leu-Asn oligomerize approximately 10 times slower than wild-type LTB [54]. As salt bridges between the A2 peptide and the last five amino acids of LTB were identified in the crystal structure of LT [55], *C*-terminal alterations to LTA and LTB may be expected to disrupt holotoxin synthesis. *In vitro* studies using a monoclonal antibody directed against the first 10 amino acids of LTB have implicated the extreme *N*-terminus of the B subunit in pentamer assembly, as well [51]. Taken together, these reports show that LTB is self-sufficient in terms of its folding and assembly, and that LTA stabilizes the holotoxin and accelerates pentamer formation.

## 4. Secretion

### 4.1. Type II secretion

Studies initially characterizing LT noted that it was found in association with aggregates in the culture supernatant that had outer membrane-like characteristics [33,56]. Several studies, in fact, described endotoxin activity and/or outer membrane fragments that were associated with LT so tightly that it complicated their attempts at purifying the toxin [33,57]. Nevertheless, early studies considered the periplasm to be the final destination for LT. However, it was also noted that LT could be secreted into the supernatant when heterologously expressed in *V. cholerae* [58]. Moreover, a mutant *V. cholerae* strain unable to secrete CT was also unable to secrete LT, indicating a common pathway for the export of both toxins [58]. Later research determined that the secretion of CT is dependent on a pathway called the type II secretion system [59]. Found in numerous gram-negative species, this system consists of a complex of 12–15 proteins spanning the inner and outer membranes [60].

In 2002, Tauschek and colleagues identified an operon in H10407 ETEC (*gspC-M*) coding for a functional type II system [61]. This system is required for the secretion of LT, debunking the apparent paradox of an extracellular pathogen producing a toxin that remains inside its own cell. Further implicating the type II system in mediating the release of LT, expression of the *E. coli* K-12 type II operon is necessary and sufficient for the export of LT from MC4100 *E. coli* cells [62]. The genes encoding the type II apparatus are also regulated by H-NS [63,64], indicating that they would be turned on under the same conditions that favor the production of LT. Furthermore, the type II system in ETEC is capable of secreting CT [65], consistent with results from the expression of LT in *V. cholerae*. 

Secretion of LT is based on the B-subunit pentamer; LTA is not a secretion-competent substrate [66]. Given the high similarity between CTB and LTB, one could expect similar mutations to impair the native secretion of each toxin. Indeed, one such mutation (E11K) that reduces the secretion efficiency of LT and CT has been found [65,67]. However, the identification of several other mutations affecting the secretion of one toxin but not the other indicates that LT and CT are recognized in different ways by their respective secretion machineries [65]. Moreover, two CTB mutants that are impaired for secretion in *V. cholerae* are secreted with wild-type efficiency from ETEC. Because these toxins are interchangeable as secretion substrates, future studies using secretion-deficient mutants could identify the portion(s) of the type II secretion apparatus that are involved in substrate recognition.

Other studies determined that the secretion of LT from ETEC strain H10407 was dependent on the activity of a protein called LeoA, which was later characterized as a GTPase [68,69]. As only 3% of strains tested in a study of numerous ETEC isolates carried the *leoA* gene [70], and Δ*leoA* mutants demonstrate pleiotropic effects [68], the role of LeoA in the secretion of LT is certainly not a universal one. ATPase activity is required for type II secretion, and it is possible that H10407 uses LeoA to provide additional energy for the export of LT. 

### 4.2. Secretion via outer membrane vesicles

The existence of extracellular LT was also explored in a second series of studies. Two reports characterized the secretion of active LT in association with outer membrane vesicles (OMVs) released by ETEC [71,72]. OMVs are spherical structures approximately 50–200 nm in diameter, composed of protein and lipid, that are released from all gram-negative bacteria studied to date [73] (A.J. Kulp and M.J. Kuehn, *Annu. Rev. Microbiol.*, in press). As the name suggests, OMVs are derived from the outer membrane, but periplasmic components are also present within their lumens. In recent years, OMVs have been shown to play a role in the virulence associated with a number of pathogens [74]. In the case of ETEC, active LT is found both inside OMVs and associated with their surface [71]. The basis and implications of this association will be discussed below.

## 5. Ganglioside Binding

### 5.1. LT’s interaction with G_M1_

Three decades ago, the host receptor for CT was determined to be the monosialoganglioside G_M1_ (Galβ3GalNAcβ4(NeuAcα3)Glcβ1-ceramide), with LT later shown to bind the same receptor [75]. When the crystal structures of CTB and LTB bound to G_M1_ pentasaccharide or galactose were compared, it was noted that the residues contacting the terminal galactose sugar in G_M1_ are conserved between the two toxins [76], explaining their similar affinities for the host receptor. The binding pocket for G_M1_ is located at the interface of two adjacent subunits, with Gly-33 providing the sole contribution of the second LTB monomer. Only B-subunit pentamers, and not monomeric CTB or LTB, are able to bind G_M1_ [77,78]. With five B subunits, there are five G_M1_ binding sites available in LTB. Whereas CTB has been modeled with five G_M1_ molecules bound [79], only one or two wild-type G_M1_ binding sites are required for internalization and toxicity of CT, albeit with slower kinetics [80]. It is not yet clear whether the same is true for LT.

**Figure 3:**
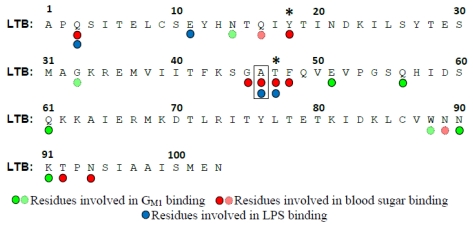
The mature polypeptide sequence of LTB, with residues involved in binding to G_M1_ (green circles), blood sugars (red circles), and lipopolysaccharide (blue circles) indicated. Lighter shades of circles indicate residues coordinating solvent molecules only. Amino acids involved in binding to G_M1_ (specifically, the terminal galactose moiety) and A-group blood sugars were identified by crystallization studies [28,76]; residues involved in lipopolysaccharide binding are inferred based on the binding phenotypes caused by mutating specific amino acids [65,86]. Asterisks indicate Tyr-18 and Thr-47; classical CTB contains a His at position 18, and El Tor CTB variants contain an Ile at position 47. These substitutions are thought to explain the lack of blood sugar binding by CTB [28]. Ala-46 (boxed) is mutated to Glu in porcine LTB, likely explaining its lack of binding to blood sugars [87]. Numbering of the LTB sequence starts with the first residue in the mature protein, after cleavage of the *sec*-dependent signal sequence.

The terminal galactose residue of G_M1 _ forms the most contacts with LTB and CTB, whereas only slight interactions with the sialic acid residue of the ganglioside have been observed [79]. The significant contact between LTB and the terminal galactose sugar, 79% of which is buried within the binding pocket, likely explains the strong preference for binding G_M1_ over other gangliosides (see Section 5.2). The LTB residues involved in G_M1_ binding are indicated in Figure 3. 

Consistent with the model presented in Figure 2, the rabbit ileum is enriched in G_M1_ compared to other sections of the small intestine [81]. Binding to G_M1_ that is located in lipid rafts on host intestinal cells is critical for the canonical pathway leading to LT’s toxic effects through increased cAMP levels. As such, the binding pocket has become an attractive target for chemical inhibitors, and several studies have involved screening galactose-based small-molecule libraries for possible receptor antagonists [82,83]. After G_M1_ binding, LT is internalized and undergoes retrograde transport to the endoplasmic reticulum [23]. There, LTA is able to retrotranslocate into the cytosol to reach its target, G_sα_. While an increasing number of studies are focused on the events surrounding LT after it enters the host cell, host effects and intracellular trafficking are not the focus of this review. Indeed, the intracellular transport of CT has been reviewed elsewhere in this special issue [84]. Ganglioside binding has also been shown to be important for the adjuvant properties ascribed to LTB and CTB. Excellent reviews of the use of CT-like enterotoxins as adjuvants are available elsewhere [85], so this property will not be discussed here either.

### 5.2. Additional non-G_M1_ substrates

The structural requirements of CT’s receptor binding seem rather strict; G_M1_ is the only ganglioside that CTB binds to with any substantial affinity [88]. Due to the existence of several hydrogen bonds contacting the terminal galactose of G_M1_, substitution of another sugar in this position is not tolerated by CTB or LTB [76]. However, a number of studies have shown that LTB can bind with low affinities to additional gangliosides, including one lacking a sialic acid residue (asialo-G_M1_), one with a second sialic acid residue (G_D1b_), and others containing a terminal *N*-acetyllactosamine disaccharide (e.g., paragloboside) [88,89] (Table 1). The structural basis of this difference in binding is not entirely clear. There is some disagreement in the literature about the ability of LTB to bind G_M2_, which lacks the terminal galactose residue present in G_M1_, and the ability of CTB to bind G_D1b_ [75,88,89,90] (Table 1). LT binding to G_M2_ was found using gangliosides immobilized on microtiter plates and thin layer chromatography assays [89,90]. Using G_M2_-containing liposomes as substrates for surface plasmon resonance, no LT binding was seen [88]. Given the extensive binding of the terminal galactose sugar in the crystal structures of CT and LT [76,79], it is difficult to imagine G_M2_ binding being important *in vivo*. Another study showed that linoleic acid, an unsaturated fatty acid found in bile, could competitively inhibit G_M1_ binding by LT [91]. However, the exact nature of this binding event is unknown, and the authors concluded that normal amounts of bile would not provide sufficient linoleic acid to impair LT’s toxicity *in vivo*.

**Table 1:** Summary of the structures of the gangliosides bound by CTB and LTB and the relative binding affinities of those interactions. ++++, strong binding; +, some weak binding; -, no binding; +/-, conflicting reports of weak binding and no binding (see text for references). Gal = galactose, GalNAc = *N*-acetylgalactosamine, Sia = sialic acid (*N*-acetylneuraminic acid), Glc = glucose, GlcNAc = *N*-acetylglucosamine, Cer = ceramide.

Ganglioside	Structure	LTB binding	CTB binding
**G_M1_**	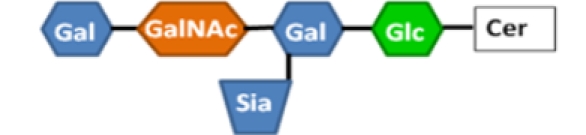	**++++**	**++++**
**Asialo-G_M1_**	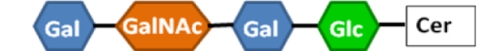	**+**	**-**
Paragloboside	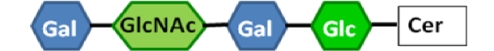	**+**	**-**
G_D1b_	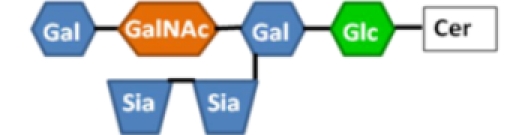	**+**	**+/-**
G_M2_	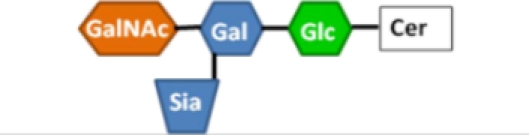	**+/-**	**+/-**

The physiological role of binding to non-G_M1_ gangliosides is currently undefined, although paragloboside can act as active receptor for LT in the rabbit intestine [89]. Paragloboside binding is largely restricted to LTB from porcine isolates, which contains three amino acid substitutions compared to human LTB (S4T, A46E, and E102K) [87]. However, human ETEC isolates sometimes carry a version of LTB identical in amino acid sequence to porcine LTB [92], and therefore, the relevance of this binding to human infection should not be completely dismissed. Binding to alternative gangliosides is of much lower affinity than LTB-G_M1_ binding (with *K_D_* values 10–25 times higher than that of G_M1_ binding), so it is easiest to imagine transient binding to non-G_M1_ gangliosides. That is, they may represent suboptimal substrates that allow LT to remain proximal to the surface of a host cell until it encounters G_M1_ and becomes internalized. 

## 6. Blood Sugar Binding

### 6.1. LTB binds to A-type blood sugars

For many years, researchers noticed that there were many more receptors for LT on the surface of the intestinal mucosa than receptors for CT and that a significant population of these receptors could not be bound by CT, indicating that they were not G_M1_ [81]. While the presence of additional ganglioside substrates for LT is certain to contribute to these observations, an increasing pool of evidence pointed toward blood antigens providing these extra binding sites. First, LT was observed to bind best to the brush borders of pigs with type A blood (with no blood type-dependent binding observed for CT) [93] and to human erythrocytes with A or B glycolipids [94]. These results were confirmed using rabbit intestinal membranes, for which the ABH blood determinants were actually functional receptors, leading to G_M1_-independent fluid accumulation [95]. Later, blood group A glycoconjugates on cultured human intestinal cells were shown to provide functional additional receptors for soluble LT [96,97]. 

### 6.2. Residues of LTB involved in blood sugar binding

**Figure 4:**
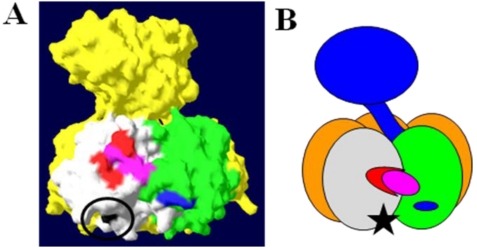
Peripheral sugar-binding pockets on LTB. (**A**) A rendering of the molecular surface of LT, with residues involved in blood sugar binding colored red, residues implicated in LPS binding colored blue, and residues involved in both binding events colored magenta. These residues lie on the interface between two B subunits, colored white and green. For reference, the galactose moiety of G_M1_ is colored and circled in black in (A) and depicted with a black star in (B). The image was rendered in Swiss-PDB viewer (version 4.0.1) using PDB number 1LTA. (**B**) A schematic representation of the subunit structure rendered in (A).

In 2007, LTB was co-crystallized in association with type-2 A group blood antigen pentasaccharide (GalNAcα3(Fucα2)Galβ4(Fucα3)Glcβ) [28]. Higher-affinity blood sugar binding had originally been observed for an artificially created LTB/CTB hybrid molecule [98], but the crystal structure demonstrated that wild-type LTB also binds blood antigen sugars. Blood antigen binding is based on a peripheral binding pocket, distinct from the residues involved in G_M1_ binding [28] (Figure 3 and Figure 4). A series of residues at positions 44–47 provides the majority of the observed protein-sugar contacts, along with Gln-3 from an adjacent subunit [28]. Consistent with the crystal structure, several point mutations involving critical residues in LTB (Q3K, Y18A, A46D, and T47A) abolish binding to the A group terminal trisaccharide, as determined by ELISA [86]. Porcine LTB contains a Glu residue at position 46 in place of the Ala residue in human LTB (see Figure 3). Because the A46D mutation in LTB abolishes blood sugar binding [86], it is likely that the A46E substitution explains the lack of porcine LTB binding to A-type glycolipids [87]. 

Binding to B-type blood sugars, which feature a terminal galactose in place of the *N*-acetylgalactosamine found in A sugars, is supported by LTB [94], but evidence from a study using the LTB/CTB hybrid indicates that binding to a closely related type-1 A blood antigen is not possible [99]. The type-1 A antigen only differs from the type-2 molecule that can be bound by LT in the nature of the linkage between two terminal sugars, so it would seem that perturbing the orientation of the sugar molecules in the blood antigen even slightly can disrupt this low-affinity binding event.

While the blood sugar-binding site is physically distinct from the G_M1_ binding site, several of its residues are directly adjacent to residues involved in G_M1_ binding (see Figure 3). One study reported that incubation of LTB with the terminal trisaccharide from A-type blood antigen does not inhibit G_M1 _binding [86]. However, another study showed that G_M1_ oligosaccharide could inhibit LTB binding to the larger A9 type-2 glycolipid substrate [100]. Given the proximity of the residues involved in each binding event, it is possible that both binding events do not (and cannot) occur at one LTB-LTB interface, but binding to G_M1_ and blood sugars may be able to occur at different locations within the B-subunit pentamer.

### 6.3. ETEC infection and blood type

The severity of *V. cholerae* infection is linked to blood type. Specifically, patients with O blood type are more likely to develop severe symptoms than their counterparts with A, B, or AB blood type [101]. In contrast, the effect of blood type on ETEC infection is much less clear [102], but a recent cohort study of ETEC-based diarrhea in Bangladesh found an increased prevalence of the disease among children with A or AB blood type [103]. Complicating the search for a correlation between ETEC infection and blood type is the existence of the so-called “secretor” phenotype. ABH blood type determinants are found in large quantities on intestinal cells, but some patients also release these glycoconjugates in their intestinal mucosal secretions [104,105]. Thus, extracellular A-type sugars may serve as decoys for LT binding in individuals with the secretor phenotype, thus protecting them against the toxin. In contrast, non-secretor patients of A blood type may in fact have additional functional receptors for the toxin. Future studies in which patients with ETEC-based diarrhea are screened for both the secretor phenotype and blood type will provide information regarding the possible use of blood group sugars as receptors *in vivo*.

## 7. Lipopolysaccharide Binding

### 7.1. Binding of LT to the *E. coli* surface is due to an association with lipopolysaccharide

**Figure 5:**
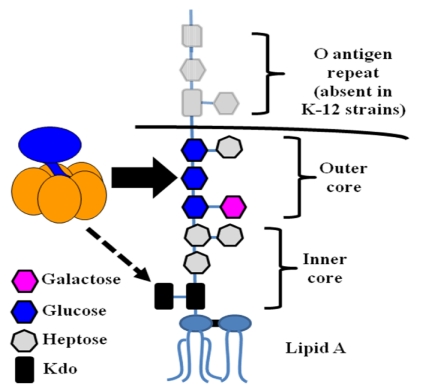
The structure of *E. coli* LPS. LPS consists of a characteristic lipid moiety (Lipid A) anchored in the outer leaflet of the outer membrane, followed by a series of sugar residues making up the inner and outer cores. In wild, non-laboratory strains (including ETEC), further oligosaccharide repeats known as the O antigen are present. Experimental evidence indicates that LT binding is mediated primarily through contacts with the outer core, with binding to Kdo possible if LPS is truncated (see below). In this diagram, the core structure of K-12 *E. coli* (such as DH5α) is presented.

In addition to its ability to bind host gangliosides and blood sugars, further research has shown that LT binds to lipopolysaccharide (LPS) on the surface of *E. coli* cells [62]. LPS is the predominant component of the outer membrane of gram-negative bacteria, consisting of a characteristic lipid moiety, Lipid A, linked to a series of sugar residues [106]. This oligosaccharide chain can be further divided into an inner core of 5–6 sugars, an outer core of 4–5 additional sugars, and the O antigen, an oligosaccharide motif repeated up to 50 times. Figure 5 shows the structure of *E. coli* LPS.

The discovery of LT binding to LPS explains a number of prior observations, including the surface-bound population of toxin molecules detected on ETEC OMVs [71]. Further experiments demonstrated that exogenously added LT can bind the surface of LT-deficient strains of ETEC [62,86]. This surface association is not dependent on the presence of the A subunit and is slightly enhanced by prior protease treatment [62]. These results indicate that the binding substrate for LTB is not likely to be a protein and that degradation of outer membrane proteins may in fact reveal more toxin binding sites.

A series of preincubation experiments determined that soluble LPS significantly inhibits the binding of LT to the surface of an LT-deficient ETEC strain [62]. Moreover, LPS lacking O antigen was equally effective as full-length LPS, if not more so, suggesting that the core sugars of LPS were the target of LT binding (Figure 5). Preincubation with Lipid A, the lipid portion of LPS, or several other bacterial phospholipids had no effect on the surface binding of LT. A later study reported that the presence of the innermost LPS sugar, 3-deoxy-D-manno-octulosonic acid (Kdo), is the minimal requirement for LT binding, but that a full oligosaccharide core is preferred [107]. 

Interestingly, while CT is able to bind to the surface of ETEC cells, neither LT nor CT binds to the surface of *Vibrio* cells [107]. Along the same lines, *E. coli* LPS inhibits the binding of CT and LT to the ETEC surface, but *Vibrio* LPS does not inhibit the ETEC surface binding of LT [107]. Thus, differences in the LPS structures of these two species of bacteria are likely to be responsible for the observed binding phenotypes. Among several differences, Kdo residues are phosphorylated in *Vibrio*, and the expression of a Kdo kinase in *E. coli* inhibits the surface binding of LTB [107].

Beyond its composition, the presentation of LPS seems to be critical for binding by LT. It has been noted that LT does not bind to LPS adsorbed to microtiter plates [100]. However, LT binds readily to the surface of *E. coli* cells [62,86] and to LPS reconstituted into liposomes (D.L. Rodriguez and M.J. Kuehn, unpublished data). Therefore, it seems that proper orientation of the LPS molecules (and, perhaps, the presence of multiple, side-by-side LPS molecules) is important for LT binding. As LT has a relative low affinity for blood sugars compared to G_M1_ [99], it is distinctly possible that the LT-LPS interaction, which involves several of the same residues (see Section 7.2), is also of low affinity. LPS adsorbed to the bottom of a microtiter well may not present its sugar residues freely enough to allow for significant LT binding. Differences in LT binding to the ganglioside G_M2_ have also been noted based on whether the substrate was present in liposomes or directly adsorbed to microtiter plates (see Section 5.2).

### 7.2. LTB residues involved in LPS binding

Several experiments indicated that LPS binding is due to residues outside the G_M1_-binding pocket. G_M1_ does not inhibit the binding of LT to the surface of bacteria, and LPS does not inhibit LT’s G_M1_-dependent toxicity in cell culture [62]. Moreover, LT is able to mediate the internalization of OMVs from a non-toxic strain of *E. coli* into cultured enterocytes in a G_M1_-dependent manner [108]. 

Upon identification of the binding pocket responsible for the association between LTB and blood group sugars, several key coordinating residues were mutated and confirmed to be required for blood group binding [28,86] (see Figure 3 and Section 6.2). Given the location of the blood group on the periphery of the LTB pentamer, distinct from the G_M1_ binding site, these mutants were also tested for binding to the surface of *E. coli* cells. Indeed, several mutations that affect blood sugar binding also reduce LPS binding [86] (Figure 3 and Figure 4). One mutant, T47A, demonstrates a particularly strong effect on surface binding to K-12 *E. coli* and H10407 ETEC cells. However, the residues involved in LPS binding are not identical to those involved in blood sugar binding (Figure 3 and Figure 4). For instance, mutation of Glu-11, a residue just outside the blood sugar binding pocket, abolishes LPS binding but not blood sugar binding [65]. Consistent with the overlapping functions of these peripheral sugar binding pockets, preincubation of LT with blood group A trisaccharide blocks subsequent binding to the surface of *E. coli* cells [86].

The T47A mutant demonstrates near wild-type levels of binding to Kdo, despite its strong impairment in binding to LPS with a full core or ETEC LPS containing an O antigen [86]. Furthermore, LTB carrying an E11A mutation does not bind to cells expressing only Kdo, but can bind to the full K-12 core present on DH5α (our unpublished data). Therefore, it seems that the sugar(s) bound by LT in the context of full-core LPS is/are something other than Kdo, while Kdo binding is possible when the sugar becomes available due to LPS truncation. Residues in the peripheral sugar-binding pocket (including those that no longer bind LPS when mutated) make extensive contacts with glucose and substituted galactose residues in A-type blood sugars [28]. As glucose and galactose are present in all known *E. coli* LPS cores [109], but not those of *Vibrio* [110], it is tempting to speculate that they may represent the binding substrate(s) of LTB.

It has been noted that classical CTB can also bind to *E. coli* LPS [107], indicating that the binding pocket is likely conserved between the two toxins. Indeed, CTB shares the residues implicated in LPS binding by LTB (see Figure 3). However, El Tor CTB contains a substitution at Thr-47 (marked with an asterisk in Figure 3), replacing the original residue with an Ile residue. This change is remarkably similar to the T47A mutation that effectively abolishes LPS binding [86], and therefore, El Tor CTB may not bind *E. coli* LPS. However, the binding of El Tor CTB to LPS has yet to be assessed.

### 7.3. Possible roles of LPS binding

There are a number of possible functions for the LPS binding exhibited by LT. Modulation of toxin level is one obvious result of the LT-LPS interaction. In comparison to freely secreted CT, more LT remains associated with the bacteria producing it. Conceivably, this interaction reduces the number of individual toxin molecules that could enter different host cells. However, the association of LT with LPS generates OMVs laden with LT molecules, inside and out. As LT can mediate the internalization of entire vesicles (and LT within OMVs contributes to toxicity) [108], these toxin-rich “bombs” may intoxicate host cells much more readily through a single binding event. Moreover, the association between LT and LPS is robust [71]; association of LT with OMVs may protect the toxin from proteolysis. The association with ETEC LPS may also prevent soluble LT from futile binding to commensal *E. coli* cells.

Another possible role for the LT-LPS interaction is in altering the host response to LT, LPS, or both. LT can lead to the intracellular trafficking of OMVs, which may modulate the host response to vesicle components. OMV-associated LT has been shown to intoxicate host cells [108], and it is becoming clear that the activation of downstream host response pathways by ETEC OMVs is not identical to the response generated by soluble LT (H.J. Chutkan and M.J. Kuehn, unpublished data). Further research on the host response to soluble and OMV-bound LT will shed light on the functional utility of LPS binding. Lastly, LT may serve as an adhesin molecule, linking ETEC cells to host G_M1_. However, given the importance of LT’s catalytic activity in promoting ETEC adherence to enterocytes [13], a function for LT as an adhesin seems less likely. Research using ETEC expressing T47A mutant toxin (which demonstrates very little surface binding) will provide additional insight into the role of the LT-LPS interaction.

## 8. Conclusions

The B subunit of heat-labile enterotoxin presents astonishing economy of amino acid use. A total of 103 amino acids make up mature LTB, with a number of them buried in its two α-helices and handful of β-strands [111]. With the residues that remain surface exposed, LTB binds the A2 helical peptide tail from LTA and forms a pentameric ring, contacting one additional B subunit on each side. In addition to a pocket for G_M1_ binding, two overlapping peripheral sugar-binding pockets enable LTB to interact with blood sugars and LPS. LTB also likely makes contacts with one or more components of the type II secretion system to be recognized for export from ETEC. Given the wide variety of binding events, it is not surprising that a single point mutation in LTB has the potential to disrupt multiple processes (e.g., T47A, which disrupts LPS and blood sugar binding, or E11K, which impairs toxin secretion and LPS binding) [65,86]. 

**Figure 6:**
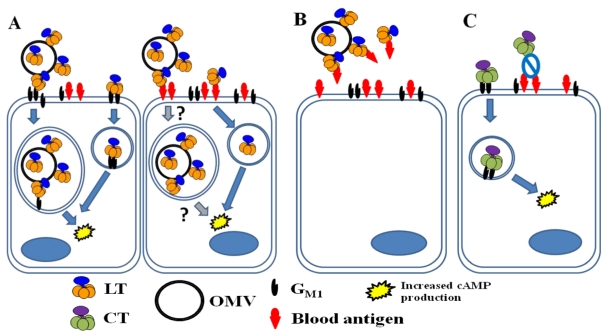
LT and CT binding in the intestine of a host with A blood type. (**A**) In a non-secretor host, LT is secreted largely in association with ETEC OMVs (both within the OMVs and on their surface). Soluble and OMV-associated LT molecules bind to G_M1_ (left cell). After internalization of an intact OMV or LT molecule, fluid and electrolyte loss occurs due to the toxin’s enzymatic activity. Because blood antigens are not present in intestinal secretions, A-group blood sugars likely also serve as functional receptors for soluble LT (right cell). Blood sugars may also be able to allow for the internalization of OMVs with surface-bound LT (indicated with question marks). It is not known whether soluble and OMV-associated LT traffic through different pathways or whether the route of trafficking depends on the nature of the receptor (G_M1_ *vs.* blood sugar). (**B**) In a host with the secretor phenotype, A-group blood sugars in intestinal secretions may serve as decoy binding substrates, limiting fluid and electrolyte loss due to LT. (**C**) CT is freely secreted by *V. cholerae* and binds only to G_M1_ on host intestinal epithelial cells, causing toxic effects after internalization.

Recent research has highlighted a few key differences between CT and LT during infection. For one, LT’s association with LPS generates a situation in which a majority of secreted LT is found in the form of toxic ETEC OMVs (Figure 6). In contrast, CT’s inability to bind *Vibrio* cells leads to the secretion of soluble CT. Moreover, the toxic effects of LT may not be G_M1_-specific, given the likelihood that blood group glycolipids of A (and possibly B) type serve as functional receptors for the toxin *in vitro* and *in vivo*. To date, the relevance of blood sugar binding has not been characterized *in vivo*. Specifically, it has not been shown that blood sugars act as a viable alternative receptor alongside G_M1_ during ETEC infection, and it is unclear whether LT is trafficked differently after internalization via blood sugar receptors. 

Research on blood sugar binding by LT in cell culture or *in vivo* has exclusively used soluble toxin, although a large portion of secreted LT is associated with LPS on the surface of OMVs. As the LPS and blood sugar binding pockets overlap significantly (see Figure 3), one cannot assume that soluble LT and OMV-bound LT will behave identically *in vivo* with regard to blood sugar binding. Due to the presence of five binding pockets per holotoxin, LPS binding and blood sugar binding may occur simultaneously in the context of OMV-bound LT, but this possibility has not yet been investigated. Therefore, studies of the association between blood type and ETEC disease must consider the possible complications of LPS binding. Furthermore, comparisons of soluble LT and OMV-associated LT may need to account for the role of blood sugar binding in the context of actual infection. Since the last comprehensive review of LT’s structure and function [17], a number of studies have provided us with new information about its regulation, secretion, and binding properties. However, there is much still to be learned about this remarkable virulence factor.
